# Bovine respiratory syncytial virus outbreak reduced bulls’ weight gain and feed conversion for eight months in a Norwegian beef herd

**DOI:** 10.1186/s13028-016-0190-y

**Published:** 2016-01-25

**Authors:** Thea Blystad Klem, Hans Petter Kjæstad, Eiliv Kummen, Hallstein Holen, Maria Stokstad

**Affiliations:** 1 Department of Production Animal Clinical Sciences, Norwegian University of Life Sciences, P.O. Box 8146 Dep, 0033 Oslo, Norway; 2 Geno Performance Test Centre, 2636 Øyer, Norway

**Keywords:** Bovine respiratory disease outbreak, Weight curve, Cost estimation, Cattle, Fattening bull

## Abstract

**Background:**

Cost-benefit evaluation of measures against respiratory disease in cattle requires accounting with the associated production losses. Investigations of naturally occurring respiratory infections in a herd setting are an opportunity for accurate estimates of the consequences. This article presents estimates based on individual monitoring of weight and concentrate intake of several hundred bulls previous to, during and after a respiratory infection outbreak with bovine respiratory syncytial virus (BRSV) as the main pathogen. The aim of the study was to analyse the association between exposure to BRSV, weight gain and feed conversion rate, quantify any change in these parameters, and estimate the duration of the change in production.

**Results:**

A comparison of growth curves for the bulls that were present during the outbreak revealed that bulls with severe clinical signs had a clear and consistent trend of poorer growth rate than those with milder or no signs. The weight/age-ratio was 0.04–0.10 lower in the severely affected bulls, and evident throughout the study period of 8 months. A comparison of growth rates between apparently healthy bulls being present during the outbreak and a comparable group of bulls exactly 1 year later (n = 377) showed a reduced growth rate of 111 g/day in the first group. The difference amounted to 23 extra days needed to reach the reference weight. Feed conversion was also reduced by 79 g weight gain/kilogram concentrate consumed in the outbreak year.

**Conclusion:**

This study indicates significant negative effects on performance of animals that develop severe clinical signs in the acute stage, and that the growth and production is negatively affected many months after apparent recovery. In addition, the performance of apparently healthy animals that are exposed during an outbreak are severely negatively affected. The duration of this decrease in production in animals after recovery, or animals that have not shown disease at all, has not previously been documented. These losses will easily be underestimated, but contribute significantly to the costs for the producer. The findings emphasize the importance of BRSV infection for profitability and animal welfare in cattle husbandry. The study also illustrates that utilising intra-herd comparison of health and production parameters is a productive approach to estimate consequences of an outbreak.

## Background

Bovine respiratory disease is amongst the most significant causes of health problems and reduced welfare and profitability in the cattle industry. Decreases in production and profitability are associated with factors such as reduced growth rate, treatment costs and increased mortality. In dairy herds, one may also observe reduced milk production, milk quality and reproductive performance [1–5].

Bovine respiratory syncytial virus (BRSV) is an important etiological agent in bovine respiratory disease [6]. The infection can be subclinical, or result in mild to severe clinical signs. Disease might be caused by BRSV directly or in combination with secondary bacterial infections which occurs frequently [7, 8]. The morbidity in a BRSV outbreak is reported to be between 60 and 80 %, and the mortality between 0 and 20 % [6].

Many experimental studies have been conducted to describe the clinical and pathological consequences of BRSV infection [7, 9–13]. However, experimental infection usually results in less severe disease compared to natural outbreaks, fewer animals are usually included, and the animals are monitored for a relatively short period after virus exposure. Studies on natural BRSV outbreaks [14–19] often lack relevant information on the situation prior to the outbreak, and suitable control groups are usually not available. Furthermore, such studies often provide little information on the production after the outbreak.

Large scale epidemiological studies from data records investigating production losses and economic impact of bovine respiratory disease are usually based on clinical diagnosis, without specific information about the etiological agent [1, 2, 4]. For BRSV specifically, reduced milk yield [5, 20] and reduced semen quality [21] are amongst the reported effects. Studies on negative consequences of this infection, relevant for rearing of young stock, are scarce and represent an important knowledge gap in the estimation of the total consequences of BRSV.

The Norwegian cattle population is currently free from several globally important respiratory pathogens such as bovine herpes virus type 1 [22] and bovine viral diarrhoea virus [23], and *Mycoplasma bovis* has never been detected [24]. This makes the Norwegian cattle population a suitable population for studying the impact of BRSV. The prevalence on herd level is estimated to be between 34 and 41 % [25]. BRSV has been found to be the main cause of outbreaks of respiratory disease, either acting as a single agent or in combination with other pathogens. Other viral pathogens known to cause respiratory disease in Norwegian cattle, are bovine corona virus (BCoV) and bovine parainfluenza virus type 3 (BPIV3) [26].

To calculate the cost-efficiency of preventive strategies for BRSV, accurate estimates of the potential losses are essential. The aim of the present study was to provide robust estimates of such losses by analysing the association between exposure to BRSV, weight gain and feed conversion rate, quantify any reduction in these parameters, and estimate the duration of decreased production. Furthermore, it was an objective to illustrate a method for estimating the effect of disease outbreaks in beef herds by employing intra-herd comparisons based on health and production records.

## Methods

The research herd belonged to an artificial insemination test centre housing up to 330 Norwegian Red bulls under standardized conditions enabling registration of a number of phenotypic traits including weight gain and body conformation. Five times throughout the year, new bulls enter the test centre. At arrival, they are about 3 months old and they stay until a maximum age of 13 months. The bulls’ age at arrival is similar to what is common in conventional Norwegian rearing units. The bulls’ health is closely monitored by the resident veterinary surgeons. The centre has loose housing pens holding 15–25 animals. The animals are grouped in pens according to their age. There are computer‒controlled concentrate feeders dispensing a concentrate formula containing 13.6 % protein and 6624 kJ per kg (Formel Favør 90, Felleskjøpet Agri, Norway). The allotment starts at 2500 g per day, and reaches a maximum of 4000 g at the age of 210 days. The concentrate consumption and body weight of the animal are recorded each time it enters the feeder. Grass silage is supplied ad libitum. The daily weight gain of the bulls in the centre is normally 1300–1400 g per day between 150 and 330 days of age.

Starting on 24 January 2011, clinical signs of respiratory disease were observed in the herd. During the outbreak period, approximately three quarters of the bulls showed clinical signs of respiratory disease to some degree. At the time of the outbreak, isolation of the sick animals was not possible. The clinical signs of disease ranged from nasal and ocular discharge, coughing, polypnea (respiratory rate ≥60 movements/minute), fever, depression and anorexia to grunting with open mouth breathing and froth, stretched neck and death. Those showing decreased uptake of concentrate, weight loss, depression and strained respiration were given a standard treatment of 2 days of meloxicam injections and five daily injections of procaine penicillin to prevent secondary bacterial infections. Of the 265 bulls present at the onset of the outbreak, 14 (5.3 %) either died or had to be euthanized because of severe respiratory distress. At the time of the outbreak, about half of the bulls had been vaccinated with a respiratory pathogen vaccine including BRSV (Bovilis Bovipast RSP vet).

### Diagnostic sampling

Serum, nasal swabs and broncheoalveolar lavage (BAL) samples were collected from seven animals in the early phase of the disease, 2 days after the onset of the outbreak. Two weeks later, a second serum sample was collected from each of the animals except one that had died. The age at the first sampling was on average 143 days.

An indirect ELISA detecting serum antibodies to BRSV, BPIV3 and BCoV (SVANOVIR^®^ BRSV-Ab, PIV3-Ab and BCV-Ab, Svanova Biotech AB, Uppsala, Sweden) was used, following the manufacturer’s instructions. In brief, the optical density (OD) reading of 450 nm was corrected by the subtraction of OD for the negative control antigen, and percent positivity (PP) was calculated as (corrected OD/positive control corrected OD) × 100. A sample was considered positive for antibodies if PP ≥ 10 and negative if PP < 10. The animals were regarded as acutely infected with BRSV, BCoV or BPIV3 if the animals seroconverted, i.e. if the first sample was negative and the second sample was positive or if they had a strong increase in antibodies, i.e. if the PP increased at least 70 %.

Real time polymerase chain reaction (RTqPCR) for detection of the N-protein of BRSV in the nasal swabs and BAL samples was performed as previously described by Klem et al. [26].

Five of the six bulls with paired serum samples either seroconverted (four) or had a strong increase in BRSV antibody level in percent positivity of at least 70 % (one). Two of the six bulls seroconverted for BCoV and two of them had a strong increase in antibody level for BPIV3. The seven animals tested were positive for BRSV on nasal swabs (five) and/or BAL samples (seven) by RTqPCR (Table 1).

**Table 1:** Results of the serological and virological testings

	Serology, paired serum samples			Virology BRSV	
Bull no.	BRSV	BCoV	BPIV3	TA	BAL
1	−	+	−	−	+
2	+	+	+	−	+
3	+	−	−	+	+
4	+	−	+	+	+
5	+	−	−	+	+
6	+	−	−	+	+
7	N/A	N/A	N/A	+	+
Total no. of pos.	5/6	2/6	2/6	5/7	7/7

The serological results are based on detection of antibodies in paired serum samples. Animals were defined as positive for infection if they seroconverted (negative to positive) or if the antibody titer increased ≥70 %

*BRSV* bovine respiratory syncytial virus, *BCoV* bovine corona virus. *BPIV3* bovine parainfluenza virus type 3, + positive for infection, − negative for infection, *N/A* not applicable, *TA* transtracheal aspirate, *BAL* broncheoalveolar lavage

Post-mortem examinations were performed on four euthanized animals. Before euthanasia, the animals were in the terminal face of respiratory disease with anorexia, grunting with open mouth breathing, froth and stretched neck. Gross pathological and histological findings were recorded. For the histological investigation, sections of the lung tissue were stained with haematoxylin-eosin. Immunohistochemistry (IHC) was accomplished by use of a cryostat on frozen sections of lung tissue with anti-BRSV isotype IgG1 kappa (Nordic BioSite, Sweden) at a dilution of 1:100. In brief, the section was fixed in 10 % buffered formalin for 2 min followed by 2 min in 70 % alcohol. Inhibition was carried out with 0.05 % phenyl hydrazine in phosphate buffered saline at 37 °C for 40 min. The blocking was accomplished with N-serum from goat 1:50 in a solution of 5 % bovine serum albumin in tris-buffered saline for 20 min. Primary antibodies were diluted in 1 % bovine serum albumin in tris-buffered saline and incubated for 1 hour at room temperature (around 21 °C). The secondary antigen (Labelled polymer, HRP anti mouse) was incubated for 30 min before developing with 3-amino-9-ethylcarbazole for 20 min and counterstained with haematoxylin for 30 s. Sections stained by IHC were examined microscopically to determine the types of cells labelled for viral antigen. Lung tissue from all four animals examined for the presence of BRSV antigen with IHC was positive.

All the examined animals had a bronchointerstitial pneumonia characteristic of a BRSV-infection: the cranioventral parts of the lungs were consolidated and mucopurulent exudate was observed in the bronchus and small bronchi. The caudodorsal parts of the lungs were distended. There was emphysema, especially in the border between the consolidated cranioventral parts of the lung and the more normal caudodorsal parts. The tracheobronchial and mediastinal lymph nodes were markedly enlarged with a mottled cut surface. Microscopic lesions consisted of necrosis of the epithelium in the bronchioli and alveoli with rejection of epithelia to the lumen. Infiltration of neutrophil granulocytes and macrophages was also present in the lumen. There was hypermia and haemorrhage from capillaries into the bronchioli and alveoli. In the bronchioli, syncytial cells typical of the paramyxovirus were formed by fusion of epithelial cells and in the wall of the alveoli hyaline membranes were present.

By culturing of lung tissue from the four autopsied calves, *Mannheimia haemolytica* was identified in one, *Pasteurella* spp. in the second, and in the two remaining, no bacteria were detected.

Based on the antibody detection of paired serum samples from the six bulls, BRSV detection by PCR of the same animals, post mortem examination of four other animals and detection of BRSV by IHC of lung tissue from these four, the conclusion was that BRSV was the principal pathogen involved.

### Data analysis

Daily data on allotted concentrate and consumption, as well as body weight, were available for a total of 816 bulls being monitored during 2011 and/or 2012. The sum of registrations resulted in a data set of more than 150.000 days of bull observations. There was a considerable frequency of missing data in the set, so that statistics relating to a variable’s value on a specific date may not have included every individual in the herd at that time. Standardization of the raw data associated with the analyses in some instances further reduced the effective number of observations. Thus, the N figures given for analyses are generally lower than the nominal number of bulls.

Two different comparisons were made.

1. The performance of the bulls classified as sick (i.e. medically treated) was compared to that of the exposed, but not sick. This was to test the correlation between short-term clinical consequences and long-term performance.
2. The exposed, but not medically treated bulls were compared with non-exposed bulls (outbreak year versus next year). The aim of this comparison was to detect an eventual effect on the performance of those exposed but not deemed to be sick.

**Fig. 1:**
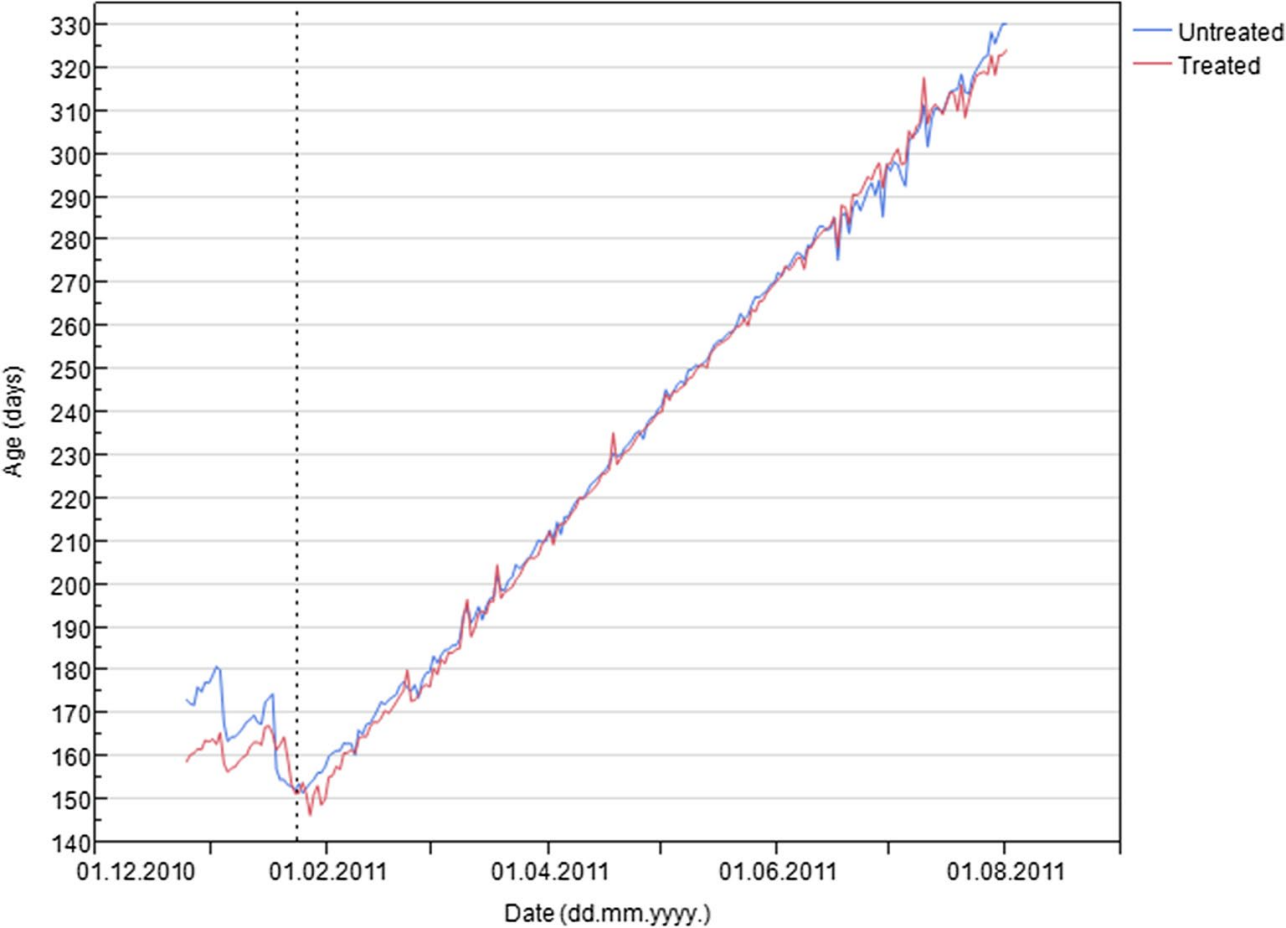
Similar age composition over time in two groups of bulls. The two *graphs* show similar* curve* shapes after excluding bulls born before a certain date (June 1, 2011) from data set. About 80 bulls were removed from a set of 220 bulls

Adjustments to the data set were necessary for statistical analyses and meaningful comparison of graphs, the main concern being the presence of age bias. For instance, the bulls getting medical treatment were on average much younger than the untreated group. To provide comparable groups for analyses involving body weight of the medically treated and untreated groups, the older bulls were excluded by adjusting the earliest included birth date downwards while watching the age distribution graphs of the two groups in the graph builder window of the statistical software. Specifically, the graphs became acceptably congruent when the bulls born after June 1 2010, i.e. younger than 230 days by January 24, 2011, remained. A plot showing the age distribution after this procedure is presented in Fig. 1. The procedure resulted in a reduction of the N value corresponding to any given day so that it led to a reduction from a maximum of 220 bulls to 143. Most of the reduction was obviously associated with the untreated group as they were of higher age. The dip in the age graphs, i.e. drop in average herd age, just before the outbreak is caused by a large cohort of young bulls being introduced at once.

To further enable comparisons of weights on the same day of animals that would not be of the same age, an age adjusted weight variable (AAW) was created by dividing an animal’s weight in kilograms by its age in days. Furthermore, two breakdown variables were created: An average daily weight gain variable (ADG) was calculated by dividing the total weight gain, i.e. the difference between the bull’s maximum and minimum body weight, by the number of days it had been housed in the centre. Secondly, a variable of the total feed conversion ratio (FCR) for each bull was created by dividing the total amount of concentrate consumed over the total monitoring time, by the total weight gain during the same period. Furthermore, in the analysis of the performance breakdown variables, ADG and FCR values based on bulls with more than 5 % missing values were deemed unreliable and excluded from statistical analysis, reducing the nominal set of bulls from 792 to 463.

The peak exposure period was defined from 2 weeks prior to and 4 weeks after the clinical onset i.e. January 10–February 4. By studying the individual weight gain curves it was possible to identify drops in body weight within this period. Each bull’s most notable drop in body weight during the 6 weeks of the highest disease incidence was identified. The lowest body weight in this drop, the highest preceding weight, the corresponding dates, and the number of days until that weight had been regained, were recorded as analysis variables.

Comparisons of group means of continuous variables were carried out employing the two-tailed t test. Investigation of associations between dichotomous variables was calculated by Fisher’s exact test (two-tailed). The statistics and analyses were performed using JMP 10 computer software (SAS Institute, Cary, NC, USA).

## Results

### Sick versus not sick bulls (present during outbreak)

Medical treatment was initiated in 56 bulls (21 %). The medically treated animals were younger than the others, their mean age at the start of the outbreak being 156 days compared to 255 days (*P* < 0.05).

The investigation of the role vaccination may have played showed that being vaccinated was highly confounded with higher age, with the vaccinated bulls being on average 27 days older than the others, and the bias was also reflected among the sick versus not sick group, the latter group being 81 days older. The bias was too pronounced to allow for control of the factor in analyses so that further quantification of vaccination effect did not provide meaningful results.

**Fig. 2:**
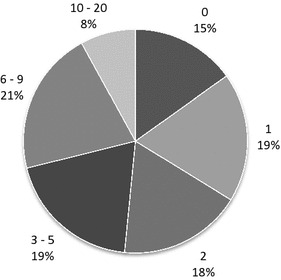
Distribution of degree of maximum drop in bulls’ individual body weight during bovine respiratory outbreak. Reduction in percent of body weight, corresponding to proportion of bulls. N = 225

A drop in body weight was seen in the majority of animals in the outbreak period. This was more pronounced in the group of the medically treated bulls (*P* < 0.05), who lost 6 % of their initial body weight while the rest had a mean drop of 3 %. Furthermore, the medically treated bulls spent a significantly (*P* < 0.05) longer period in the decrease from maximum to minimum weight (5.0 days) than the untreated (3.1 days). Figure 2 shows the distribution of animals according to weight loss.

The medically treated and untreated bulls consumed the same amount of concentrate (as  % daily ration), during the peak infection period, the maximum difference being only 0.26 percentage points.

**Fig. 3:**
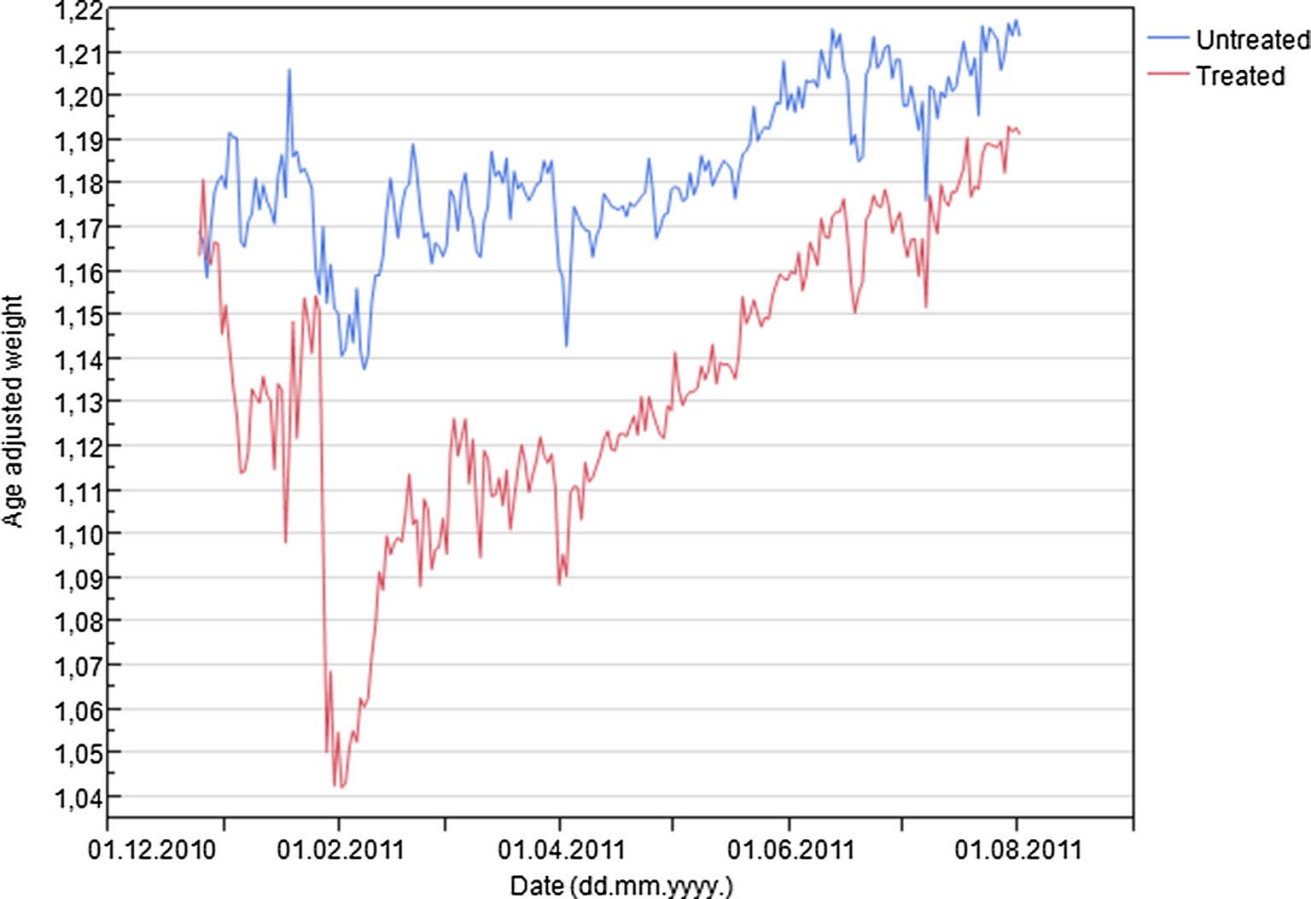
Weight to age ratio in two groups of bulls present during outbreak. Bulls that were diagnosed and treated for respiratory disease versus those without diagnosis and treatment. The figure shows the ratio in relation to the outbreak date 24 January 2011

A plot of the two groups’ AAW over time revealed a consistent difference in disfavour of the medically treated bulls. The difference developed quickly around the peak outbreak date, the 24 January 2011, reaching its maximum just after. The trend was evident from the date of the outbreak and was then visible for several months (Fig. 3). Statistical analyses of AAW on selected dates confirmed the trend and provided estimates of the difference in AAW of 0.04–0.10 (Table 2).

**Table 2:** Age adjusted body weights in sick bulls versus not sick bulls following respiratory disease outbreak January 24th

Date (days after onset of outbreak)	Feb. 1 (7)	Mar. 25 (60)	May 25 (120)
N^a^	106	143	138
Not treated (n)	1.14 (64)	1.18 (85)	1.19 (82)
Medically treated (n)	1.04 (42)	1.12 (58)	1.15 (56)
Difference	0.10**	0.06	0.04

** *P* < 0.05

^a^The figure varies from day to day because of occurrence of missing values from the automated weighing

### Exposed versus non-exposed bulls (year versus year comparison)

The general disease incidence numbers per year showed a clear difference in the incidence of respiratory disease between the outbreak year and control year (Table 3).

**Table 3:** General health status of bulls in outbreak year versus control year

Year	2011	2012
Animals at risk	543	516
Drug treatment for respiratory disease	64	26
Drug treatment for other disease	93	96
Euthanasia/death from respiratory disease	14	0
Euthanasia/death from other disease	0	0
Emergency slaughter	1	7

**Fig. 4:**
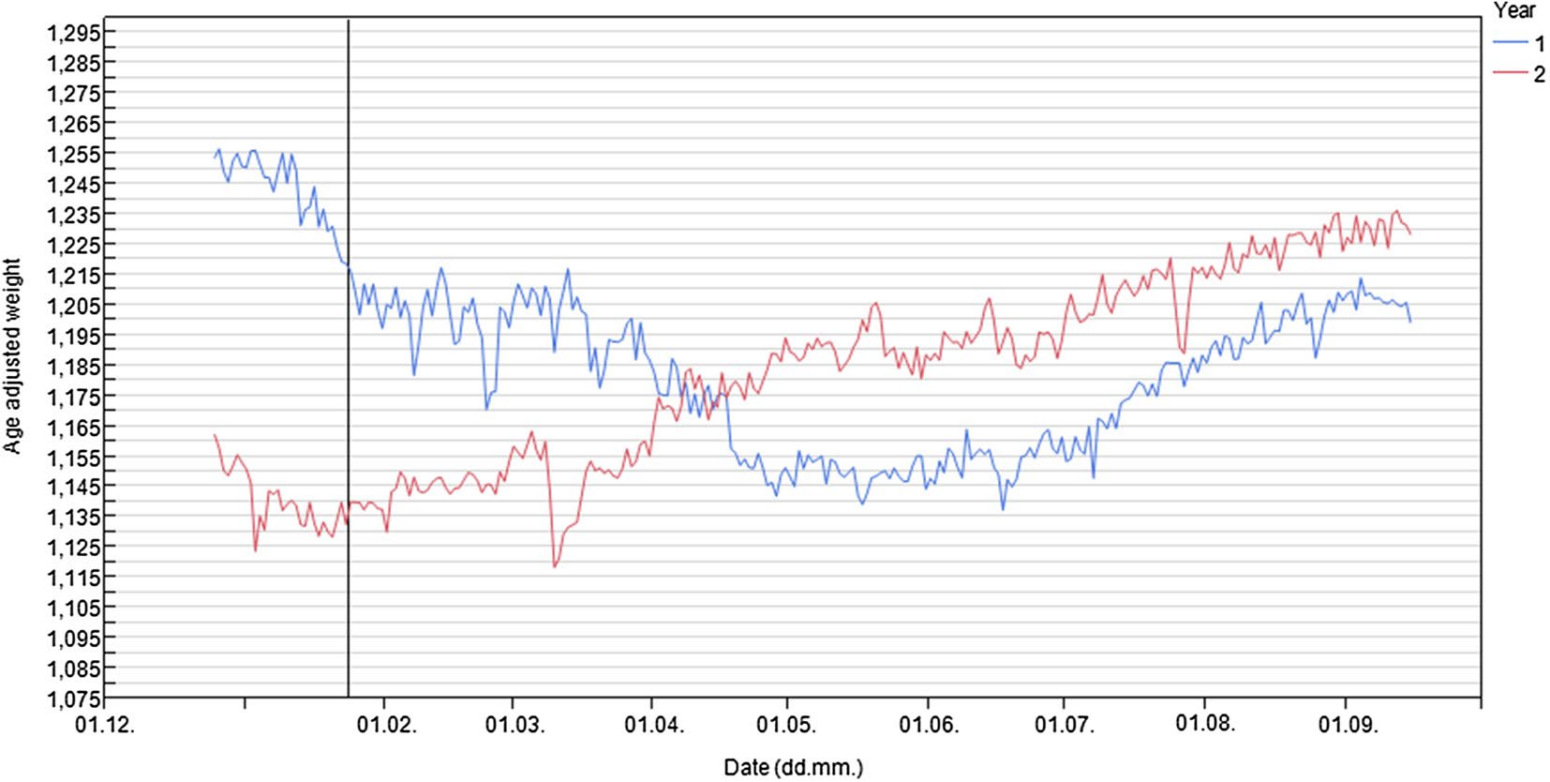
Weight to age ratio in two groups of bulls, year versus year comparison. Comparison of bulls without a diagnosis of respiratory disease monitored in the test centre during 2011 (Year 1) versus 2012 (Year 2). Respiratory disease outbreak date in 2011 is denoted by *vertical line*

The plot of mean AAW over time, comparing 2011–2012 date by date (Fig. 4), showed that the AAW of 2011 started higher, then fell to a lower level and stayed below the control year level for many months. When the groups were compared with respect to average FCR as well as ADG, the 2011 group had poorer figures (Table 4).

**Table 4:** Average daily weight gain (ADG) and ADG/amount of concentrate consumed in respiratory disease outbreak year versus following year

	ADG	ADG
		1000 g concentrate consumed
Following year (n = 177)	1255	423
Outbreak year (n = 128)	1144	345
Difference	111*	78*

* P < 0.05

## Discussion

Even though there were indications of other respiratory pathogens present in the herd, it is highly likely that BRSV was the main cause of the outbreak. Firstly, the clinical signs and the high number of cases in a short period of time indicate a highly virulent infectious agent being the likely cause of the outbreak investigated in the present study. Serological examination did indicate BRSV infection in most animals. Even if increasing antibody levels to BCoV and BPIV3 were also detected, the increase was present in a lower number of animals. In addition, BRSV was detected by RTqPCR. Furthermore it is reported that BPIV3 acting as a single agent mainly causes mild disease, and even when coinciding with BRSV infection it does not change the clinical signs significantly [27, 28]. Similarly, respiratory BCoV infections usually cause mild disease in infected calves [29]. Infection with BRSV predispose to infection with opportunistic bacteria [7], and it has been reported that 40 % of the viral infections lead to secondary bacterial infections [8]. In Norway, *Pasteurella multocida* and *Trueperella pyogenes* are the two most common bacteria causing secondary infections. *Mannheimia haemolytica* are also relatively commonly detected and *Histophilus somni* appears to be rare [30, 31]. Primary pulmonary pasteurellosis is generally uncommon in beef or dairy cattle in Norway, and it has never been diagnosed in the centre described in the present study. The severity and character of the outbreak indicated a secondary origin of the bacteria cultured from lung tissue. BRSV as the principal pathogen in the outbreak is also supported by the detection of pathological lesions typical for BRSV infection and the IHC with positive staining of BRS viral antigen. The losses connected with the outbreak may be due to either primary effect of the virus and/or secondary bacterial infections.

At first, the analysis of vaccination status seemed to indicate a protective effect of vaccination. However, because the age distribution between the two groups with respect to experiment variable as well as outcome variable (medical treatment) was highly unequal, it is not possible to draw this kind of conclusion. Higher age would be associated with, i.e., generally stronger physique, more acquired immunity from factors outside of vaccination, and more time for the vaccine to have produced any effect. The data thus are not suitable for this type of analysis.

A main finding of the present study is the clear effect on the growth and concentrate feed conversion ratio, and that the effect is present in not only the medically treated and untreated group, but also when pooling all animals from the outbreak year compared to the next. The latter finding indicates that the outbreak affected a far larger group of animals than just those deemed to be in need of medical treatment.

Growth rate is amongst the most important factors for the profitability of rearing of cattle as it determines the slaughter weight and feed costs [32, 33]. Reduced feed conversion obviously represents an economic loss *per se*, and will exacerbate the negative effect of reduced growth rate when present at the same time.

The significant and quick drop in body weight seen in many animals during the outbreak occurred with no corresponding drop in concentrate consumption, not even in the treated bulls. This indicates that the drop was caused by a reduced intake of roughage and/or water in the initial phase of disease and that even sick bulls manage to finish their concentrate. It is significant in the sense that sick cattle usually have a decreased appetite, so that a normal concentrate feed consumption may falsely be interpreted as a sign of good health unless one also considers other observations.

Except for a 3-week period of the outbreak, the total amount of roughage consumed in the present population was reported to be as normal.

The findings emphasize the economic significance of BRSV infection. As an illustration of the clinical significance of the differences concerning the AAW variable, a bull with an AAW of 1.20 at 300 days of age would be 30 kg heavier than a bull of the same age with an AAW of 1.10. At 150 days, the same AAW difference corresponds to 15 kg. Similarly, a reduced daily weight gain from 1255 to 1144 g (Table 4), will over 8 months amount to 23 extra days needed for the more slowly growing bull to reach the same weight. For similar rearing systems, these numbers may be multiplied with the economic figures appropriate for a specific country or industry, to produce cost estimates.

Some considerations of factors associated with the studied herd are important for interpreting the results correctly. At any moment there are animals of different ages and weights being housed in different areas throughout the building. Furthermore, removal of older and introduction of new bulls occurs regularly. Finally, there is a possible effect of season as longitudinal fluctuations occur in ambient temperature, roughage quality, staff composition, and stocking density. In the present study it was attempted to control for such factors, firstly by ensuring equal age distribution between the groups compared. Furthermore, when comparing bulls that had been housed at different times of the year, the comparison was made by using year as the grouping variable, ensuring that any seasonal influence would be similar across the two groups. However, the herd composition was not fully comparable from 2011 to 2012, or the graphs in e.g. Fig. 3 would have been more congruent and eventually differed only in level. Early 2011 had an influx of an unusual large group of young test bulls. A full adjustment for age differences was not possible and this may have influenced the statistics for the first part of the year. However, in the following months this effect fades as the curves attain similar shapes.

The research herd stocked animals of the same sex and of the same breed inside the same building where they were fed the same diet. The recording of weights and feedings were automated, thus likely to have provided consistent, comparable and precise data. The animals were also monitored by the same stockmen and veterinary surgeons. Thus, the disease cases are similarly likely to have been assessed and treated in a consistent manner from case to case. These conditions made the herd exceptionally well suited for a scientific study, i.e. standardized to a degree difficult to find in other herds.

To provide even more robust comparisons, it would have been preferable to use more years than just one for comparison with the outbreak year. Preceding years, e.g. 2010 were considered, but these years were also problem years with high frequency of respiratory disease cases, which was in fact the reason why the initial research project was started. Those years may not have made a meaningful contrast and were therefore abandoned. Even earlier years were not readily available from the test centre’s recording system and shortly after 2012, weight gain was no longer one of the phenotypic test variables. The quality control of this variable was then less rigorous and it would consequently not be as suitable for analysis. However, the value of adding several seasons of comparisons also has its limits. Pooling too many years would tend to compound a lot of variation from many sources into an average that may or may not represent a good base for comparisons. Our comparison year has a well-documented health status and is close to the outbreak year, which ensures that herd level factors have had less time to change. The true size of the effect of the outbreak in the study herd is arguably underestimated by our analyses. The higher number of deaths in the outbreak year compared to the control year is one cause. These cases could obviously not be fully included in analyses or graphs, but had they lived, their data would undoubtedly have emphasized the poor performance of their group. Furthermore, the available bull level disease information was in the form of a dichotomous treatment variable, which certainly will classify the sickest animals correctly but probably missed some bulls with milder clinical signs. Nevertheless, these bulls may also show decreased performance parameters. On the other hand, genetic progress would in itself contribute to higher performance the next year, which produces an opposite estimation bias. However, the increase in growth rate in this cattle population is well documented and amounts to a reduction of 1 day in a bull’s time to slaughter [34], which is on a lower order of magnitude than the differences found in the present study and therefore not very important to the discussion.

The absolute values of performance parameters such as growth rate, weight, and feed conversion are influenced by management, breed, feeding as well as numerous other factors that may be particular to the research herd. Many of those specific factors can probably be summarised as better than average management, housing, feeding and health care, all contributing to less severe consequences of airway infection than in commercial herds. Thus, the exact numbers do not necessarily apply directly under other circumstances, but the general trends in the results as well as the findings of differences between groups in the present study have a higher degree of validity and should be generally applicable.

## Conclusions

The present study shows that intra-herd comparison of health and production data can be a valuable approach to estimate the costs associated with reduced health status. More specifically, the study shows that BRSV affects the growth performance of bulls for several months after infection. The effect is most pronounced in animals that are seriously affected during infection, but a significant loss of performance can also be detected in animals showing mild or no signs of disease, and despite apparent recovery. This amounts to a significant part of the costs associated with BRSV, however they may easily be underestimated. Such losses are of great concern to the farmer and must be taken into account in any cost-benefit analysis of preventive measures towards BRSV infection, whether on herd or population level.
